# Determinants of quality of life in primary family caregivers of patients with advanced cancer: a comparative study in southern China

**DOI:** 10.3389/fpubh.2023.1034596

**Published:** 2023-05-24

**Authors:** Jiaqi Lin, Zhuoxin He, Guanhua Fan

**Affiliations:** Shantou University Medical College, Shantou, China

**Keywords:** home-beased hospice care in China, family caregivers, patients with advanced cancer, hospice volunteer service, China

## Abstract

**Objective:**

To examine and compare the quality of life (QoL) of the primary family caregivers (PFCs) of inpatients with advanced cancer and the PFCs of home hospice patients with advanced cancer and to analyze the determinants of QoL.

**Methods:**

Four hospices and three comprehensive or tumor hospitals in Guangdong Province, China were research sites. QoL was measured using paper-based and online questionnaires. Multiple stepwise linear regression was used to analyze the determinants of QoL of PFCs.

**Results:**

The PFCs of inpatients had significantly better QoL than did the PFCs of home hospice patients (*p* < 0.01). One-way ANOVA results indicated the following: for the PFCs of inpatients, PFC age (*t* = 2.411, *p* < 0.05), type of relationship with patient (*F* = 2.985, *p* < 0.05), and family economic situation (*F* = 3.423, *p* < 0.05) significantly affected PFCs’ QoL; for the PFCs of home hospice patients, family economic situation (*F* = 3.757, *p* < 0.05) and care experience (*t* = 2.021, *p* < 0.05) significantly affected PFCs’ QoL. A multiple stepwise linear regression was conducted: for the PFCs of inpatients, family economic situation and whether the PFC was the patient’s immediate family member were included as predictors of QoL; for the PFCs of home hospice patients, family economic situation and care experience were included as predictors of QoL.

**Conclusion:**

Our findings can help improve the home hospice care service model in mainland China. In particular, the QoL of the PFCs of home hospice patients requires urgent attention. The PFCs of home hospice patients requires more nursing guidance and interactions with community.

## 1. Background

In China, the incidence of cancer has been increasing approximately 0.2% in males and 2.2% in females over the past 10 years (1), making it a major public health problem (1–3). In particular, patients with advanced cancer have constituted the majority of people who are dying (1). With greater demand for better quality of life (QoL) (3), hospice care has attracted increasing attention from both doctors and patients. Hospice care may help in addressing challenges from China’s aging population (2), and it is supported by government policies, such as *Healthy China 2030* (4). In particular, home hospice care is a model of community care service. In such care, the hospice service team, which consists of community medical staff and volunteers, provides relief and supportive care for home hospice patients and their family (5). Because the survival time of patients may be longer than their period of hospitalization (6), many end-of-life patients with cancer in China prefer to spend their final days at home rather than in the hospital (7–10). This preference has been informed, in part, by constant improvements to hospice-related policies and the hospice service model (11). This phenomenon of patients with advanced cancer favoring home hospice care has shifted the nursing responsibility from formal nursing staff to the informal caregiver (i.e., the family’s primary caregiver) (7–9). Primary family caregivers (PFCs) refer to family members (e.g., spouses, children, parents, or siblings) who live with the patient and who assume most of the nursing responsibility (12).

QoL is defined as an individual’s experience of their living conditions with respect to their goals, expectations, standards, and perception of events; such an experience differs depending on the cultural value system that the individual is part of (12). Contemporary studies have demonstrated (1) that home hospice care can improve the QoL of the PFCs of end-of life patients with cancer (13) but (2) that QoL significantly differs, in part or in whole, between patients with advanced cancer in hospital and their counterparts in hospice care (10, 13). Studies have noted the following determinants of the QoL of the PFCs of patients with advanced cancer: gender (14–17), physical health (12, 18, 19), psychological status (10, 12, 18–20), religious belief (21), family economic situation (19–20, 22–23), relationship with patient (14), cognitive evaluation of care (19, 21), care duration (24), professional support (12), family support (7), and social support (12, 19, 20, 24). Considering the aforementioned context, this study analyzed the QoL of the PFCs of patients with advanced cancer, comparing the differing influence on QoL from hospitalization versus hospice care. Other determinants of QoL were also analyzed. The purpose of this study is to elucidate problems in home hospice care mode by comparing the QoL in PFCs which are, respectively, in home hospice and in hospital, therefore the application of the hospice care service model is promoted and attention on the QoL of the PFCs of patients with advanced cancer is drawn to end-of-life medicine. We assume that determinants of QoL may differ between the care trajectory. This may reveal a source of strength of hospice and may suggest areas to improve in hospice care service in China.

## 2. Methods

### 2.1. Participants

A PFC was recruited if they satisfied all of the following criteria: The PFC should be above 18 years old, without mental or cognitive disease, able to understand the survey. Besides, the PFC should be immediate family member of the patient and bears most responsibility for patient care. The PFC has cared his/her patient more than 2 weeks without employment relationship with the patient and receives no compensation for their care. What’s more, the patients with advanced cancer in this study all met the basic standards for receiving hospice care, including pathological diagnosis, survival assessment for less than 3 months, and active choice of palliative care. When the participant has any employment relationship with the patient, or mental disease, or difficulty to communicate, or the participant is reluctant to continue the survey, the PFC would be excluded.

With reference to the design standard of the international scale, the sample size is calculated by 5–10 times the number of variables. In order to ensure the stability and reliability of the research results, this study takes 10 times the number of variables of the scale (our quality-of-life scale is considered here). Based on this, it was expanded by 20% to offset the lack of sample size caused by invalidation. Finally, the sample size required by this study was about 180 (assuming that the quality-of-life scale was about 15 variables). Geographically, hospitals and hospice centers are distributed in various locations in Guangdong Province, which can better represent South area of China. Institutionally, the three hospitals are tertiary general hospitals, tertiary specialist hospitals and secondary general hospitals, representing hospitals of different levels，and the four hospice centers selected were the only four hospice centers in Guangdong Province at that time of the study.

### 2.2. Materials

Every participant completed a questionnaire in paper or electronic form. The questionnaire inquired into (1) demographic information on participants and their patients and (2) PFC’s QoL. The scale was based on the World Health Organization Quality of Life scale (WHOQOL questionnaire) (25), both its general version and its WHOQOL-BREF Taiwan version; the Taiwan version was simplified from the WHOQOL and created from Taiwan’s community life form (26). The scale comprised 13 questions. The questionnaire was adopted after double-blind translation. Responses to all the questions were scored on a five-point Likert scale, and positive or negative scores were assigned to a response depending on the content of the question. Finally, the sum of all scores for each question constituted the participant’s total QoL score (maximum score: 65, indicating the highest QoL). A hospice volunteer team from Shantou University Medical College was authorized to conduct a pre-investigation when they visited patients in home hospice care. After the reliability and validity of the questionnaire were statistically verified, the formal study was conducted in Guangdong Province, China. Before the formal survey, the questionnaire was sent to several experts who have been engaged in hospice practice and research for a long time in tumor hospitals and hospice canters. After their review, the rationality of the questionnaire design can be reconfirmed.

### 2.3. Procedure

Because of differences in research objects and places, the PFCs of inpatients and those of home hospice patients were surveyed differently.

#### 2.3.1. PFCs of inpatients

Three comprehensive or tumor hospitals in Guangdong Province were selected as main research sites: Shenzhen Hospital of Southern Medical University, Shantou Longhu Hospital, and Cancer Hospital of Shantou University Medical College. The researchers visited tumor wards and hospice wards. Subsequently, with the recommendation and assistance of the department head, the PFCs of inpatients with advanced cancer in the ward were administered the electronic questionnaire in person; the questionnaires were accessed by scanning a QR code. Participants who completed the questionnaire truthfully and passed the quality inspection of the questionnaire were compensated for their time through Internet payment. However, to respect patient privacy and avoid disrupting their treatment, PFCs’ personal data were only collected after consultation with the respective hospital’s ethics committee.

#### 2.3.2. PFCs of home hospice patients

Four hospice centers in Guangdong Province were selected as main research sites. Paper and electronic questionnaires were administered. Researchers visited the hospice center of the First Affiliated Hospital of Shantou University Medical College, which was where the first hospice center in mainland China was established. With permission by the hospice center, a paper-based questionnaire survey was administered to PFCs who were unable to scan the QR code to access the electronic questionnaire. Participants who completed the questionnaire truthfully were compensated for their time with daily necessities, such as detergent, diapers, and paper towels. For those who could scan the QR code, they were able to complete the survey online and were compensated for their time through Internet payment. The survey was conducted online for the hospice centers of Affiliated Hospital of Guangdong Medical University, Shenzhen People’s Hospital, and Chaozhou Central Hospital. We contacted the staff of these hospice centers for their assistance in distributing our survey through their official communication channels. The returned questionnaires were screened for their suitability.

### 2.4. Ethical assessment

Prior to the administration of the formal surveys, this study was approved by relevant ethical approval and those of each hospice center and hospital approved this study. We also chose hospice centers as our research site to minimize disruptions to home hospice patients and to reduce the large cost of door-to-door visits. A hospice center is where family members of home hospice patients obtain medication and seek treatment, which allowed us to communicate with PFCs in person. Furthermore, prior to each survey, we described the purpose and content of the questionnaire to the participant and requested their informed consent before proceeding. During the field investigation, the researchers accompanied the participants and answered the participants’ questions in real time. If a participant exhibited psychological distress, we suspended the survey, attempted to comfort them, waited for them to regain their composure, and then asked if they wished to continue the survey.

### 2.5. Statistical analysis

To ensure that data were valid, the collected questionnaires were screened according to whether the participant satisfied all inclusion criteria and whether they answered the questionnaire completely. The questionnaire data were input into Excel 2016 and analyzed using SPSS software (Version 23.0, SPSS, Chicago, IL, United States). The mean value and standard deviation were used as descriptive statistics. The higher the score, the higher the QoL of PFCs. The questionnaire’s Cronbach’s *α* coefficient was acceptable at 0.780. KMO = 0.81 > 0.6, and *p* < 0.001 in Bartlett’s test of sphericity, indicating the questionnaire qualified for factor analysis. Prior to parametric testing, normality of dependent variable was assessed using Shapiro–Wilk’s normality test and homogeneity of variances was assessed by Levene’s test. The determinants of QoL were analyzed using the chi-square test, independent sample *t* test, one-way ANOVA, and multiple stepwise linear regression.

**Figure 1:**
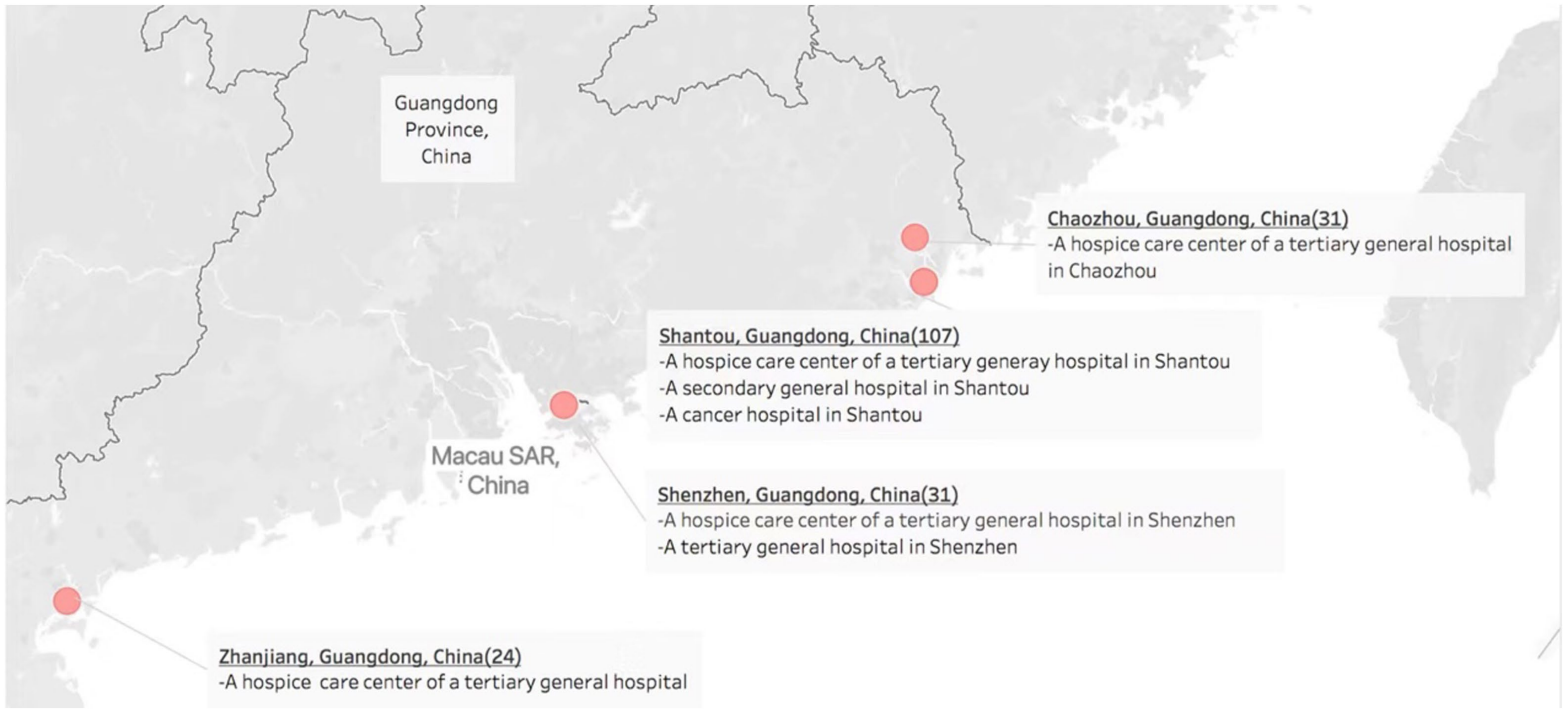
Distribution of oncology wards and hospice centers in four cities in Guangdong, China. Note: OpenStreetMap is a collaborative map of the world that creates a world map. Anyone can contribute to OpenStreetMap, and OpenStreetMap’s data is free to share and use. The Mapbox Streets tileset contains data derived from OpenStreetMap.

Figure 1 showed the distribution of selected oncology wards and hospice centers in Guangdong, China.

## 3. Results

### 3.1. Sociodemographic characteristics of sample

In total, a total of 232 questionnaires have distributed in this study and 193 valid questionnaires were collected in this study, among which 88 were from the PFCs of inpatients and 105 were from the PFCs of home hospice patients. After screening, 167 valid questionnaires were included for analysis (effective rate: 86.5%), among which 67 were from the PFCs of inpatients (effective rate: 76.1%), and 100 were from the PFCs of home hospice patients, with an effective rate of 95.2%. Tables 1, 2 summarize the characteristics of the PFCs and home hospice patients, respectively. According to the results of the chi-square test and Fisher’s exact test, the PFCs of inpatients and the PFCs of home hospice patients significantly differed with respect to the education level, family economic situation, and whether a nursing worker was employed.

**Table 1:** Characteristics of participating PFCs.

Characteristics	Inpatient (*N* = 67)	Home hospice (*N* = 100)	*p*
	Number (%)	Number (%)	
Gender			0.296
Male	32 (47.8)	56 (56.0)	
Female	35 (52.2)	44 (44.0)	
Age, year, mean (SD)	46.06 (14.56)	41.84 (14.38)	0.021^c^
Educational level			0.046^c^
Elementary or below	8 (12.0)	21 (21.0)	
Secondary	34 (50.7)	58 (58.0)	
Post-secondary^a^	25 (37.3)	21 (21.0)	
Marital status			0.355
Single	16 (23.9)	15 (15.0)	
Married	48 (71.6)	75 (75.0)	
Divorced	1 (1.5)	5 (5.0)	
Widowed	2 (3.0)	5 (5.0)	
Religion			0.079
No religion	36 (53.7)	41 (41.0)	
Chinese folk religion	17 (25.4)	18 (18.0)	
Buddhism	9 (13.4)	32 (32.0)	
Islam	0 (0)	2 (2.0)	
Christian	1 (1.5)	2 (2.0)	
Catholicism	2 (3.0)	1 (1.0)	
Taoism	1 (1.5)	1 (1.0)	
Other	1 (1.5)	3 (3.0)	
Type of relationship			0.968
Adult child	33 (49.2)	46 (46.0)	
Spouse	17 (25.4)	24 (24.0)	
Sibling	4 (6.0)	9 (9.0)	
Parents/children-in-law	7 (10.4)	9 (9.0)	
Grandparent or grandchildren	2 (3.0)	5 (5.0)	
Other	4 (6.0)	7 (7.0)	
Daily care duration			0.385
0–4 h	15 (22.4)	17 (17.0)	
5–8 h	10 (14.9)	10 (10.0)	
9–12 h	19 (28.4)	26 (26.0)	
More than 12 h	23 (34.3)	47 (47.0)	
Total care duration			0.083
2 weeks to 1 month	10 (14.9)	5 (5.0)	
1 to 3 months	13 (19.4)	22 (22.0)	
3 to 6 months	14 (20.9)	15 (15.0)	
More than 6 months	30 (44.8)	58 (58.0)	
Family economic situation^b^			0.001^d^
Surplus	7 (10.4)	4 (4.0)	
Break-even	29 (43.3)	28 (28.0)	
In debt	22 (32.9)	64 (64.0)	
Unclear	9 (13.4)	4 (4.0)	
Have care experience	28 (41.8)	48 (48.0)	0.430
Caring for others while caring for patient	32 (47.8)	56 (56.0)	0.296
Have family members who take turns rendering care	52 (77.6)	68 (68.0)	0.176
Nursing worker employed	12 (17.9)	7 (7.0)	0.030^c^
Living with patient	55 (82.1)	72 (72.0)	0.134

a Post-secondary includes college, university, and junior college.

b Answering this question was optional out of respect for the participant’s privacy.

c *p* < 0.05.

d *p* < 0.01.

**Table 2:** Characteristics of home hospice patients.

Characteristics	Number (%)	Range
Gender		
Male	58 (58.0)	
Female	42 (42.0)	
Age, year, mean (SD)	64.45 (12.17)	30–91
Marital status		
Single	3 (3.0)	
Married	85 (85.0)	
Widowed	7 (7.0)	
Other	5 (5.0)	
Housemate at home, number, mean (SD)	4.43 (2.29)	1–15
Hospice duration, months, mean (SD)	7.16 (6.89)	1–41
Primary site of cancer		1–3^b^
Lung	38 (38.0)	
Other	21 (21.0)	
Liver	15 (15.0)	
Colorectum	12 (12.0)	
Cervix	5 (5.0)	
Breast	5 (5.0)	
Esophagus	4 (4.0)	
Stomach	4 (4.0)	
Nasopharynx	4 (4.0)	
Lymph	2 (2.0)	
Thyroid	1 (1.0)	
Metastasis site of cancer		1–5^c^
Skeleton	28 (28.0)	
Lung	27 (27.0)	
Other	26 (26.0)	
Lymph node	25 (25.0)	
Liver	24 (24.0)	
Cerebrum	11 (11.0)	
Spinal cord	9 (9.0)	
Symptom		1–11^d^
Pain	93 (93.0)	
Dyspnea	18 (18.0)	
Nausea and vomiting	39 (39.0)	
Constipation	47 (47.0)	
Edema of lower limb	32 (32.0)	
Ascites	12 (12.0)	
Lymphatic edema	11 (11.0)	
Asthenia	49 (49.0)	
Insomnia	36 (36.0)	
Gatism	9 (9.0)	
Other	7 (7.0)	
Consciousness status		
Conscious	69 (69.0)	
Dazed	11 (11.0)	
Delirious^a^	7 (7.0)	
Semi-comatose	10 (10.0)	
Comatose	3 (3.0)	

a Delirium includes disturbance of consciousness, behavioral confusion, and inattention.

b The range that number of primary sites of cancer.

c The range that number of metastasis sites of cancer.

d The range that number of symptoms.

### 3.2. Total QoL score differences between the PFCs of inpatients and the PFCs of home hospice patients

According to the independent sample *t* test for differences between both the groups of PFCs, the PFCs of inpatients had a higher QoL score than did the PFCs of home hospice patients (Table 3).

**Table 3:** Total QoL scores of the PFCs of inpatients and the PFCs of home hospice patients.

Type of patient	Total score (Mean ± SD)	*t*	*p*
Inpatient	34.84 ± 6.25	−2.708	0.007^a^
Home hospice	31.90 ± 7.25		

a *p* < 0.01.

### 3.3. Analysis of QoL determinants

Comparing the demographic data of the PFCs of inpatients with those of the PFCs of home hospice patients (Table 4), we noted that the QoL of the PFCs of inpatients significantly differed with respect to the relationship with patient, PFC’s age, and family economic situation (*p* < 0.05) but not with respect to gender, educational level, and PFC care experience (*p* > 0.05). However, for the PFCs of home hospice patients, their QoL was significantly affected by family economic situation and care experience (*p* < 0.05) but not by gender, educational level, and the relationship with patient (*p* > 0.05).

**Table 4:** Determinants of QoL for the PFCs of inpatients and PFCs of home hospice patients.

Variable	Inpatient				Home hospice			
	Total QoL score (mean ± SD)	*t*	*F*	*p*	Total QoL score (mean ± SD)	*t*	*F*	*p*
Gender of PFCs		−1.613		0.112		−0.593		0.555
Male	33.56 ± 5.40				31.52 ± 6.78			
Female	36.00 ± 6.81				32.39 ± 7.85			
Age of PFCs			2.411	0.046^a^			0.495	0.779
<30	32.56 ± 4.83				34.09 ± 10.58			
30–39	32.31 ± 5.35				30.59 ± 4.37			
40–49	36.43 ± 7.75				32.70 ± 7.77			
50–59	38.71 ± 6.05				31.40 ± 6.75			
60–69	38.00 ± 5.07				30.29 ± 7.20			
≥70	37.00 ± 5.29				32.10 ± 8.28			
Educational level of PFCs			0.410	0.665			0.284	0.753
Elementary or below	36.63 ± 4.78				31.29 ± 6.17			
Secondary	34.38 ± 6.64				31.76 ± 7.13			
Post-secondary	34.88 ± 6.24				31.90 ± 8.69			
Relationship with patient			2.985	0.018^a^			0.866	0.507
Adult child	32.58 ± 4.74				32.22 ± 7.04			
Spouse	36.71 ± 6.54				31.00 ± 7.00			
Sibling	42.00 ± 2.45				31.78 ± 7.61			
Parents/children-in-law	37.71 ± 9.38				30.89 ± 7.49			
Grand child	32.00 ± 2.83				37.80 ± 8.70			
Other	34.75 ± 6.25				30.14 ± 8.15			
Family economic situation			3.423	0.040^a^			3.757	0.027^a^
Surplus	38.86 ± 5.67				36.75 ± 8.06			
Break-even	36.00 ± 6.97				34.11 ± 7.39			
In debt	32.59 ± 5.08				30.36 ± 6.93			
Have care experience		−0.055		0.956		2.021		0.044^a^
Yes	34.79 ± 7.14				33.42 ± 8.04			
No	34.87 ± 5.63				30.50 ± 6.19			

a *p* < 0.05.

### 3.4. Multiple stepwise linear regression for QoL of PFCs of inpatients

The determinants of QoL listed in Table 4 for the PFCs of inpatients were recombined to form 8 predictors, which we denoted *X*_1_ to *X*_8_ (Table 5). After verifying that the total QoL score for these PFCs was normally distributed, we chose *α* = 0.05 and 0.10 as the inclusion and exclusion criteria, respectively, for a multiple stepwise regression analysis. Variance inflation factors (VIFs) were used to diagnose multicollinearity, and predictors with 1 < VIF < 3 were retained. The predictors of *immediate family member* (*X*_4_) and *family economic condition 1* (*X*_5_) were included in the model (*F* = 7.180, *p* = 0.002), and they accounted for 17.8% of the variation in QoL (Table 6). PFCs who were immediate family members had a significantly lower QoL than did PFCs who were non-immediate family members. With regard to family economic situation, the PFCs of inpatients had significantly poorer QoL if their family was in debt than if their family income yielded a surplus or was at break-even. The multiple stepwise linear regression equation for the QoL of the PFCs of inpatients (*Y*) was as follows:


$$Y=58.129-6.805\;X_{4}+5.662\;X_{5}$$

**Table 5:** Predictors and quantified units of the QoL of the PFCs of patients with advanced cancer.

Predictors	Code	Quantified unit
Gender	*X*_1_	1: male, 2: female
Academic qualifications	*X*_2_	1: not highly educated, 2: highly educated
PFC age	*X*_3_	1: younger than 60 years, 2: older than 60 years
Immediate family member^a^	*X*_4_	1: non-immediate family member, 2: immediate family member
Family economic condition 1	*X*_5_	1: not surplus, 2: surplus
Family economic condition 2	*X*_6_	1: not break-even, 2: break-even
Family economic condition 3	*X*_7_	1: not in debt, 2: in debt
Care experience	*X*_8_	1: have, 2: do not have

a Immediate family members includes parents, spouse, and adult child.

**Table 6:** Multiple stepwise linear regression results for predicting the QoL of the PFCs of inpatients and the PFCs of home hospice patients.

Types of patients	*R* ^2^	Adj. *R*^2^	Predictors	Coefficients	Std. error	Standard coefficients	*t*	*p*	VIF
Inpatient	0.207	0.178	Intercept	58.129	6.359	0	9.142	<0.001	
			Immediate family	−6.805	2.055	−0.403	−3.311	0.002	1.029
			Family economic condition 1	5.662	2.383	−0.289	−2.376	0.021	1.029
Home hospice	0.131	0.113	Intercept	31.834	2.819	0	11.295	<0.001	
			Family economic condition 3	−4.050	1.411	0.278	2.870	0.005	1.007
			Care experience	−3.733	1.410	−0.257	−2.648	0.010	1.007

### 3.5. Multiple stepwise linear regression for QoL of PFCs of home hospice patients

The preceding analysis for *X*_1_ to *X*_8_ was also applied to the PFCs of home hospice patients (Table 5). After verifying that the total QoL score for these PFCs was normally distributed, we used the aforementioned inclusion and exclusion criteria of *α* = 0.05 and 0.10, respectively, for a multiple stepwise regression analysis. VIFs were used to diagnose multicollinearity, and predictors with 1 < VIF < 3 were retained. The predictors of *family economic condition 3* (*X*_7_) and *care experience* (*X*_8_) were included in the model (*F* = 7.039, *p* < 0.001), and they accounted for 11.3% of the variation in QoL (Table 6). Similar to the PFCs of inpatients, the PFCs of home hospice patients had significantly decreased QoL if their family was in debt. Furthermore, PFCs with care experience had higher QoL compared with those with no care experience. The multiple stepwise linear regression equation for the QoL of the PFCs of home hospice patients (*Y*) was as follows:


$$Y=31.834–4.050\;X_{7}–3.733\;X_{8}$$

## 4. Discussion

### 4.1. Overall difference in QoL between PFCs of inpatients and PFCs of home hospice patients

In this study on end-of-life patients with advanced cancer, we found that the PFCs of inpatients had significantly higher QoL compared with the PFCs of home hospice patients. This finding is consistent with that reported by Spatuzzi et al. (13) but not with the finding reported by Rha et al. (18). Furthermore, Spatuzzi et al. (13) reported that compared with the PFCs of patients receiving active treatment, the PFCs of patients receiving home hospice care had significantly poorer mental health but better physical and general health. We surmise this difference in QoL to be due to the assistance rendered by medical staff to inpatients. Such assistance relieves PFCs’ caregiving burden and may also lend optimism to the patient’s prolonged survival. Additionally, hospital staff can provide timely intervention and professional care, which gives PFCs a sense of security. Furthermore, death is perceived to be further for inpatients than for home hospice patients, which reduces PFCs’ psychological burden; this, in turn, improves their mental health. However, with regard to physical health, the PFCs of inpatients may be exhausted by round trips between their homes and the hospital. By contrast, for the PFCs of home hospice patients, a hospice team pays regular visits to the patient’s house. Furthermore, in China, these PFCs only need to pick up their free medicine at hospice centers once every two weeks.

### 4.2. Determinants of QoL for PFCs of inpatients and PFCs of home hospice patients

#### 4.2.1. Determinants for PFCs of inpatients

For the PFCs of inpatients, the survey findings indicated that QoL was significantly affected by age, relationship with patient, and family economic status but not by gender, educational level, and care experience.

##### 4.2.1.1. Age

According to our results, for PFCs younger than 70 years, older PFCs (those in their 50s and 60s) had better QoL. However, for PFCs older than 70 years, older PFCs had poorer QoL. We attribute this result to the following. PFCs in their 50s and 60s tend to be more skilled at patient care and more open-minded toward illness and death, which reduces the burden of care (27–29) and improves their mental health (30). However, for PFCs older than 70 years, their physical health is likely to decline to a point where (31–36) patient care becomes difficult, which reduces their QoL. Similarly, Ribé et al. noted that age predicts caregiver QoL (31): PFCs of advanced age tended to have low QoL in the physical and social domains.

##### 4.2.1.2. Type of relationship

According to our results for the PFCs of inpatients, QoL was the highest if the PFC and patient were siblings and the lowest if they had a parent–child or grandparent–grandchild relationship. Our multiple stepwise regression further indicated that PFCs had lower QoL if the patient was an immediate family member than if the patient was not. We attribute this result to differences in the perceptions of patient progress between the types of PFC–patient relationships, where these differences in perceptions are due to differences in beliefs regarding disease and death at different ages. Because the PFCs of inpatients are often involved in decision making regarding the patient’s treatment plan (37), these PFC decision makers must deeply understand the patient’s disease condition, and their views on the patient’s condition will greatly affect their mood. Therefore, the type of relationship that the PFC has with the patient greatly affects their QoL. By contrast, the PFCs of patients in home hospice care are less likely to make major medical decisions and their responsibilities are limited to patient care. This explains why the relationship type did not significantly affect the QoL of the PFCs of home hospice care patients in our findings. Specifically, in this study, sibling PFCs tended to have a close relationship with the patient due to similarities in age and worldview. Thus, these siblings tended to support, comfort, and be empathetic toward each other. Parents, who are traditionally the main caregivers, may have lower QoL from taking on an excessive care burden when they are PFCs (38). In a case study of grandchildren PFCs, Boquet et al. (39) noted that these grandchildren PFCs had career and family obligations because of their age, which made caring for the grandparent especially onerous; the authors also noted that these grandchildren PFCs tended to feel guilty when they failed to render good care (39). Furthermore, in contrast to studies reporting that spousal PFCs have significantly lower QoL (10, 14, 36), our study noted no significant difference for spousal PFCs. We attribute this to differences in the definition of variables: in contrast to these other studies, we defined the relationship type to encompass a wider variety of relationships instead of only spousal relationships.

##### 4.2.1.3. Family economic situation

According to our findings and consistent with that reported by Rha et al. (18), family economic situation significantly affected QoL. Specifically, the multiple stepwise regression results indicated that QoL was significantly improved by having a family income that yielded a surplus. We attribute the results to the following. Family economic status determines the family’s ability to bear the patient’s medical expenses. For families of inpatients, their economic status directly affects which treatment plan is chosen, where less well-off families are less likely able to choose a more expensive treatment plan. Furthermore, inpatients incur a much larger medical expense than home hospice patients do, and family economic status affects the social functioning of PFCs. Zhu et al. (40) noted that relatively well-off households can hire workers to reduce PFCs’ workload, thus improving the PFC’s QoL.

#### 4.2.2. Determinants of QoL for PFCs of home hospice patients

For the PFCs of home hospice patients, the survey findings indicated that QoL was significantly affected by family economic status and care experience but not gender, educational level, and type of relationship.

##### 4.2.2.1. Family economic situation

According to our results for the PFCs of home hospice patients, QoL was improved by a better family economic situation. Specifically, the multiple stepwise regression results indicated that QoL was significantly decreased if a family was in debt. This is also a determinant of that in PFCs of inpatients. We attribute this finding to psychological stress from being unable to pay for inpatient care because of debt. Furthermore, our Fisher’s exact test results indicated that the families of home hospice patients were significantly less well-off than the families of inpatients. Thus, home hospice care was often the last resort for PFCs who had high levels of psychological stress. A participant stated the following:

*I stopped the medication because the cost was so high that my family could not afford*. *I said,* “*We can always borrow from other people and gradually pay it back*.” *But he refused*.

Woman, PFC, patient’s wife (41).

Our results for the PFCs of home hospice patients were slightly different from those for the PFCs of inpatients. We attribute this difference to more well-off PFCs having higher QoL standards, which partially biased the survey results. Although Zhu et al. (40) noted that well-off families can hire a helper to assist with patient care, the serious and deteriorating condition of their loved one negatively affects the PFC’s mental health and social life. Furthermore, in a qualitative study, McDonald et al. (42). noted that the financial care burden affects caregivers’ QoL. In general, the PFCs of home hospice patients have greater QoL if their family is more financially resilient.

##### 4.2.2.2. Care experience

**Figure 2:**
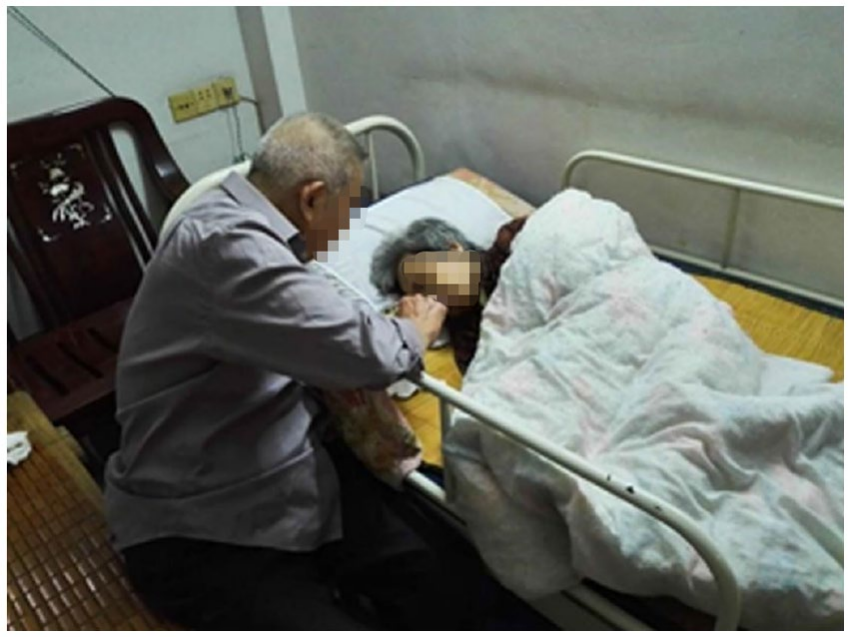
PFC care for his wife who had advanced cancer. Photographed by a medical volunteer of the hospice volunteer team of Shantou University Medical College. Faces of both were blurred for privacy.

Consistent with previous studies, our findings indicated that PFCs’ QoL increased by having nursing experience (43–45). Care experience is a determinant of QoL in home hospice PFCs that is not present in PFCs of Inpatients. We attribute this finding to the following. Under the model of home hospice care, PFCs usually undertake most of the care work (Figure 2). By contrast, for the PFCs of inpatients, such care work is often undertaken by hospital staff. Thus, care experience only significantly affected the QoL of the PFCs of home hospice patients but not the PFCs of inpatients. Furthermore, being more skilled, PFCs with nursing experience find care work to be easier, which improves their QoL. Similarly, Hao (46) analyzed the QoL of the PFCs of patients with epilepsy and noted that PFCs have greater QoL from decreased psychological stress if they are better equipped with disease-related knowledge and have better access to medical resources.

### 4.3. Other determinants

As for PFCs’ gender, many studies (14–17) have reported that female PFCs tended to have lower QoL. However, we noted higher QoL for female than for male PFCs, regardless of whether they cared for an inpatient or home hospice patient. Physical (12, 18–19) and mental health (10, 12, 18–20), as well as the cognitive evaluation of care (19, 21), have been found to affect QOL in PFCs; our study, however, did not analyze these determinants. According to previous studies, religion can predict QoL (21). However, this effect was not noted in our findings. We attribute this to most of our participants adopting their local folk religion (the worship of the deity known as *Tudigong*, the Earth God), as has been the tradition in China’s Chaoshan area. Thus, the choice to adopt such a religion was not a conscious one. However, some of our participants who rendered home hospice care noted the importance of religion in their lives:

*I’m a Buddhist myself, and I feel like this (taking care of the husband with advanced cancer) is the right thing to do*. *Even though it’s hard to take care of my husband, I should do my duty*.

Woman, 79 years old, PFC, patient’s wife. (ID No. 87).

In contrast to our study, a previous study found educational status to be a determinant of QoL (18). We attribute this difference to our limited research area. Generally, the educational level is positively related to QoL in PFCs (18).

To summarize, the PFCs of inpatients had higher QoL compared with the PFCs of home hospice patients. For the PFCs of inpatients, age, relationship type, family economic situation, and care experience affected QoL; for the PFCs of home hospice patients, family economic situation, and care experience affected QoL. Some factors were noted to be nonsignificant probably because of particularities in our research location.

## 5. Suggestions for promoting home hospice services

We propose the following suggestions. These suggestions are aimed at improving the quality of hospice care services and promoting their use for the families of patients with advanced cancer.

First of all, more basic health guidance for PFCs is needed to improve their health condition, which is the premise for them to take good care of their patients. Besides, necessary financial support is essential for poor patients with advanced cancer. In China, there are already many policies and benefits to reduce the economic burden of patients, including medical insurance, social insurance and subsistence allowance. What’s more, it can be known from the results that more than half of participating PFCs had no previous care experience, and more than 70% of PFCs had not received post-secondary education. These suggest that the PFCs of patients with advanced cancer are unlikely to have the necessary home-nursing knowledge and skills. In addition, Rha et al. found that PFCs have better QoL when their caregiving burden is reduced (18). Thus, nursing training equips PFCs with skills that allow them to care for patients with greater ease, thus improving their QoL (47). Specifically, the hospice service team can also dispatch medical student volunteers to visit homes. These student volunteers can render care to the end-of-life patient with cancer and impart care skills to PFCs, which improve the PFC’s QoL. This measure can be executed in the context of the present-day status of hospice care service in China.

## 6. Limitations

Our study has the following limitations. First, the patients under the care of the participating PFCs had varying courses and severities of the disease, which might have biased the results. Although a longitudinal study can account for such bias, it leads to a serious loss of follow-up as the limited life expectancy of patients with advanced cancer. Thus, we adopted a cross-sectional study instead. Because of this limitation, we could not further analyze differences in QoL between the PFCs of inpatients and the PFCs of home hospice patients through time-based comparisons. Besides, more longitudinal study is required to verify the relationship between these determinants and the QoL of PFCs.

Our questionnaire scale combined the WHOQOL questionnaire and the WHOQOL-BREF Taiwan version. Although the applicability of the two scales has been verified by many studies and we statistically verified the reliability and validity test of our scale, more research about hospice is required to evaluate whether this scale is suitable for promotion.

## 7. Conclusion

This quantitative comparative study of QoL and its determinants investigated the PFCs of inpatients and the PFCs of home hospice patients. After collecting and analyzing responses from PFCs, our study provides evidence that the QoL of PFCs of home hospice patients is significantly lower than that of inpatients. The QoL of PFCs of home hospice patients might be affected by family economic status and care experience, while the QoL of PFCs of inpatients might be affected by age, relationship with the patient, and family economic status. That is, although the hospice care model has been implemented in mainland China for almost 20 years, the QoL of the PFCs of home hospice patients requires urgent attention. The model of home hospice care should focus more on the PFCs of home hospice patients.

## Data availability statement

The raw data supporting the conclusions of this article will be made available by the authors, without undue reservation.

## Ethics statement

The Ethics Committee of Shantou University Medical College (SUMC-2021-54) approved the data collection procedures that involve study participants to ensure their accordance with the ethical standards. Informed consent was obtained from all individual participants included in the study.

## Author contributions

JL undertook the data analysis, completed statistical tables and result analysis, wrote method, results, suggestion, acknowledgement, and interpreted the conclusion. ZH performed literature review and data collection, made the flow chart and map, wrote the background, method, and discussion. GF was in charge of the conception design, the design of the study framework and survey questionnaire, research process, interpreted the results, and ultimately modified the manuscript. All authors contributed to the article and approved the submitted version.

## Funding

Medical research fund Project of Guangdong Province (A2021500 and A2022535)，General program of Guangdong Natural Science Foundation (2022A1515012192), Shantou Science and Technology Plan’s Medical and Health Category Project (190716185262435, SFK [2019] No. 106-10). Philosophy and Social Sciences Planning Foundation Discipline Co-Construction Project of Guangdong Province of 2022 (GD22XXW10), “Climbing Plan” Guangdong University Student Science and Technology Innovation Cultivation Project (pdjh2019b0193). Undergraduate teaching quality and teaching reform project of Guangdong Province in 2022 (Yue Gao Jiao Han[2023]4-581). Clinical Teaching Base Teaching Reform Research Project of Guangdong Province in 2021(2021JD062).

## Conflict of interest

The authors declare that the research was conducted in the absence of any commercial or financial relationships that could be construed as a potential conflict of interest.

## Publisher’s note

All claims expressed in this article are solely those of the authors and do not necessarily represent those of their affiliated organizations, or those of the publisher, the editors and the reviewers. Any product that may be evaluated in this article, or claim that may be made by its manufacturer, is not guaranteed or endorsed by the publisher.
