# Supplementary figures and images for: Determinants of quality of life in primary family caregivers of patients with advanced cancer: a comparative study in southern China
Source: Front Public Health. 2023 May 24;11:1034596. doi: 10.3389/fpubh.2023.1034596 (PMC10248401; doi:10.3389/fpubh.2023.1034596)

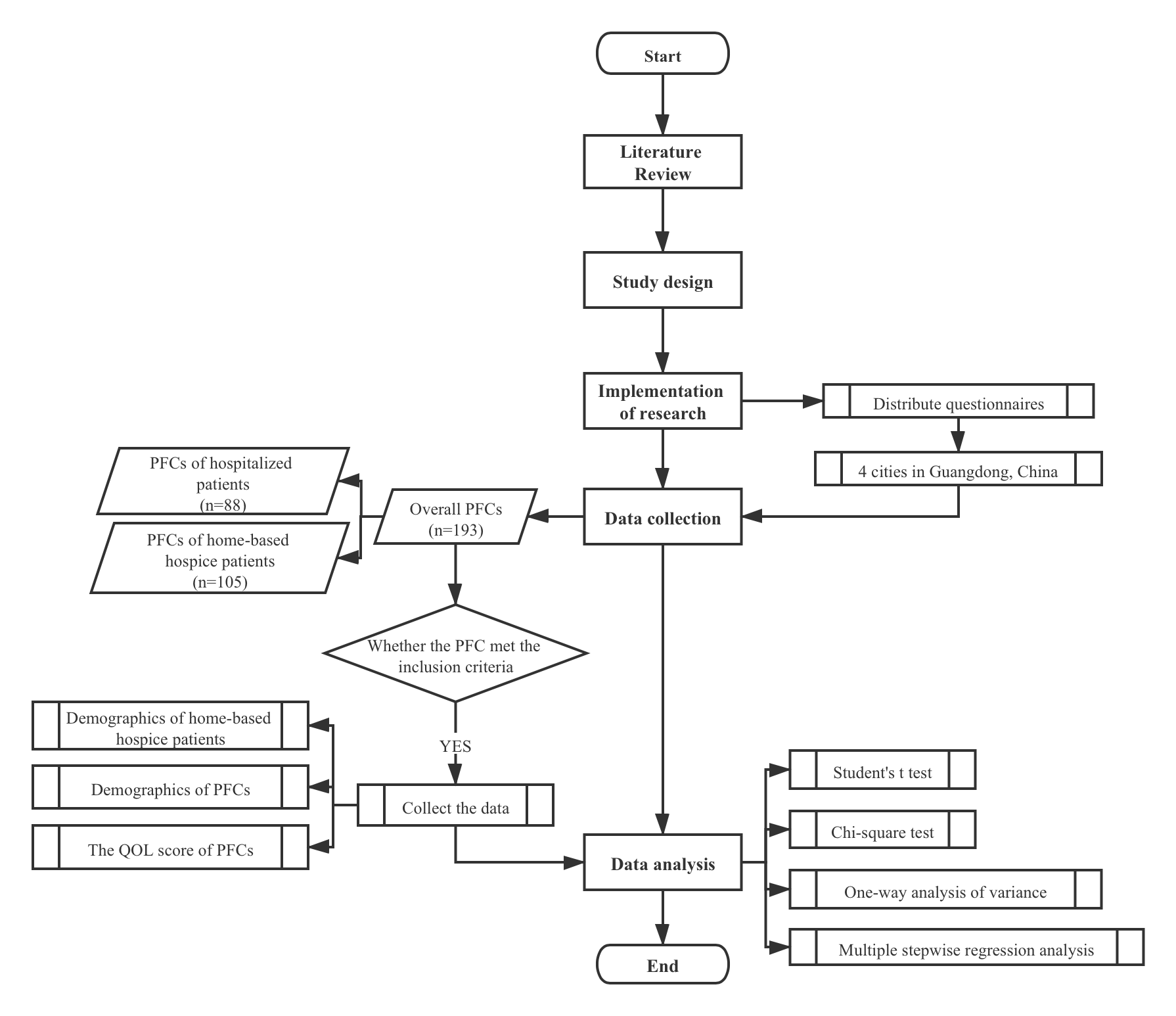

Supplement: Supplementary file 1 [file Image_1.TIF]
